# The choice of message and messenger to drive behavior change that averts the health impacts of wildfires: an online randomized controlled experiment

**DOI:** 10.1186/s12889-022-14801-6

**Published:** 2022-12-16

**Authors:** Payam Aminpour, Jennifer F. Helgeson, Paul J. Ferraro

**Affiliations:** 1 Department of Environmental Health and Engineering, a joint department of the Whiting School of Engineering and Bloomberg School of Public Health, Johns Hopkins University, Baltimore, MD 21202 USA; 2 Applied Economics Office, Engineering Laboratory, National Institute of Standards and Technology, Gaithersburg, MD 20899 USA; 3 Carey Business School, Johns Hopkins University, Baltimore, MD 21202 USA

## Abstract

**Background:**

To reduce the negative health effects from wildfire smoke exposure, effective risk and health communication strategies are vital. We estimated the behavioral effects from changes in message framing and messenger in public health messages about wildfire smoke on Facebook.

**Methods:**

During September and October 2021, we conducted a preregistered online randomized controlled experiment in Facebook. Adult Facebook users (*n* = 1,838,100), living in nine wildfire-prone Western U.S. states, were randomly assigned to see one of two ad versions (narrative frame vs. informational frame) from one of two messengers (government vs. academic). We estimated the effects of narrative framing, the messenger, and their interactions on ad click-through rates, a measure of recipient information-seeking behavior.

**Results:**

Narrative frame increased click-through rates by 25.3% (95% CI = 22.2, 28.4%), with larger estimated effects among males, recipients in areas with less frequent exposure to heavy wildfire smoke, and in areas where predominant political party affiliation of registered voters was Republican (although not statistically different from predominantly-Democrat areas). The estimated effect from an academic messenger compared to a government messenger was small and statistically nonsignificant (2.2%; 95% CI = − 0.3, 4.7%). The estimated interaction effect between the narrative framing and the academic messenger was also small and statistically nonsignificant (3.9%; 95% CI = − 1.1, 9.1%).

**Conclusions:**

Traditional public service announcements rely heavily on communicating facts (informational framing). Shifting from a fact-focused, informational framing to a story-focused, narrative framing could lead to more effective health communication in areas at risk of wildfires and in public health contexts more broadly.

**Trial registration:**

Date registered: August 19, 2021; Registration DOI: 10.17605/OSF.IO/JMWUF

**Supplementary Information:**

The online version contains supplementary material available at 10.1186/s12889-022-14801-6.

## Background

Increased temperatures and drought associated with climate change continue to affect the frequency, extent, and severity of wildfires globally [1]. In the Western U.S., for example, fire frequency and the average extent of annual burned land has increased over the last seventy years, with the average size of fires more than doubling [2]. In addition to generating deadly flames, wildfires also generate harmful smoke over large areas downwind from the fire. This widespread smoke has important public health implications for large populations of people who are not at direct risk from the fire’s flames [3].

To reduce the negative health effects from wildfire smoke, effective risk and exposure mitigation communication strategies are vital. Public health messaging strategies about wildfire smoke traditionally rely on informational messages, like public service announcements (PSAs), that are delivered by government messengers and use scientific facts to communicate the health risks associated with wildfire smoke. These conventional messages are typically presented in the form of expository or didactic texts that leverage logic and reasoning to induce behavioral change. However, this informational message framing may fail to effectively drive mitigation behavior because it traditionally uses an inaccessible language that is too broad to be useful or relevant at the individual level and too technical to understand when presented to the average person [4]. An additional challenge in motivating behavior change is a lack of public trust in government messengers [5, 6]. There is evidence that mistrust in messages conveyed by government actors may be a key factor preventing action to combat public health problems (e.g., Martin et al. [7]) and other social policy problems, such as climate change (e.g., Hoffman [8]). Recent studies suggested that experimental design can advance efforts to achieve effective messaging strategies to increase wildfire risk mitigation [9–11].

Here, in a pre-registered online randomized controlled experiment [12], we test how the behavioral effects of messaging strategies for wildfire health risks vary with different framings and the messenger types. We target digital messages to adult users of Facebook in wildfire-prone, U.S. communities. In a 2 × 2 design, we vary the message framing between a narrative (story-based) framing and an informational (fact-based) framing, and vary the messenger between an academic messenger and a government messenger.

In recent years, the use of narratives (story-based communication) has received considerable attention by scholars in the fields of science and health communications [13–15]. We define narratives in line with Kreuter et al., which is a definition frequently adopted in the public health communication literature: “Narrative is a representation of connected events and characters that has an identifiable structure, is bounded in space and time, and contains implicit or explicit messages about the topic being addressed” [16]. A nonnarrative, however, adopts “expository and didactic styles of communication that present propositions in the form of reasons and evidence supporting a claim” [16]. Both narrative and nonnarrative forms of communication can carry the same message.

To explain how narratives can be effective in persuading and engaging people, theoreticians have proposed a set of mechanisms, such as narrative transportation [17], identification [18], and emotion [19]. These mechanistic theories posit that narratives can capture an audience’s attention by transporting them into the narrative’s world and by triggering cognitive and emotional systems [14], ultimately synthesizing complex ideas into comprehendible frameworks that are central to the human experience. They can therefore be persuasive by drawing individuals into the events relayed in the narrative and by encouraging the recipient to relate to the characters within the narrative. By bringing attention to health issues through engaging plot lines and relatable characters, narratives are thought to effectively convey public health messages [20]. Additionally, when employed in risk communication, narratives have been shown to effectively induce mitigation behavior (see references in Byerly et al. [21]). Communicators may therefore use narratives in their risk mitigation-focused interventions. To our knowledge, however, these effects have not been tested in the wildfire smoke context and through evidence-based field experiments.

The effectiveness of narratives may, however, depend on who delivers them. A growing body of evidence, particularly from the field of behavioral economics, has reported that the messenger can have important implications for how audiences process a message [22–25]. Among commonly identified mechanisms of the messenger effect are the expertise and trustworthiness of the messenger, which determine the extent to which a messenger may be deemed credible and thus influence a message recipient’s attitudes or behaviors [26, 27]. Therefore, two messengers who provide the same information under identical framings can trigger different interpretations and subsequently different behavioral effects.

Despite the attention on the effects of narrative framing and messengers, experimental studies typically look at the effects of narrative framing [21, 28–31] separate from the effects of messengers [32, 33]. The individual effects of each intervention (i.e., message framing and messenger) may be modest, but the effects cumulatively add up. Yet, very few studies investigate the cumulative effect of multiple behavioral interventions in the same context, and even fewer do it in a clean 2 × 2 factorial design. Few studies explored the interaction between message framing and messenger as behavioral interventions (e.g., [26, 34]); however, they frequently suffer from several important limitations, including underpowered empirical designs, issues of multiple comparisons, and using self-reported outcome variables to evaluate treatment effects. None of those studies report on observed behavioral outcomes in naturally occurring settings.

Our experimental study uses real behavioral outcomes in a naturally occurring online setting to explore the effects of public health message frame, messenger, and their interaction within the context of wildfire smoke and through digital delivery and engagement. With a sample of over 1.8 million Facebook users, we estimated the effects of ads that manipulated message framing (*Narrative* vs. *Informational*) and messenger type (*Government* vs. *Academic*) on click-through rates (CTR: the rate at which Facebook users click on the ad after being exposed to it). When clicked, the ads transferred the user to a landing page, which provided links to outreach materials and educational resources aimed at improving public awareness about the health effects of wildfire smoke and the actions people can take to mitigate exposure. By conducting a “natural field experiment” [35], wherein subjects are exposed to treatments in a setting that is “real life” as opposed to a controlled laboratory environment, we enhanced the external and ecological validity of our study. Through our use of a natural online setting, respondents’ behavior is more likely to represent typical online behavior.

Observing real behavior associated with messaging and risk communication in natural hazards contexts can be notoriously challenging. Given these challenges, previous studies [9, 10] recommended that scholars observe information-seeking behavior as a “low-cost” and “meaningful” behavioral response. Therefore, our findings have implications for risk communicators who seek to use innovative messaging strategies for building trust and communicating effectively in public health contexts, like wildfire smoke safety.

### Health risks of wildfire smoke

Wildfire smoke is a mix of toxic gasses and particles, such as benzene, carbon dioxide, carbon monoxide, and fine particulate matter (e.g., PM_2.5_) – those particles ≤ 2.5 μm in diameter – which are known to be dangerous for humans when exposed (both in- and outdoors) [36]. These particles can pass through the nose and throat and enter the lungs, and the smallest particles can even penetrate the blood circulatory system. Smoke inhalation and exposure can increase the risk of cardiovascular- and respiratory-related effects, such as asthma attacks, pneumonia, stroke and chronic heart and lung diseases [37].

Given the adverse effect of wildfire smoke on public health, minimizing exposures to mitigate these health effects is of paramount importance. Effective communication can influence smoke-prone communities to engage in risk-reduction behaviors, [38, 39] including information-seeking and planning [9, 10] for wildfire events ahead of time that will further mitigate the health risks of exposure.

## Methods

We conducted an online message-framing experiment through Facebook ads. We used a 2 × 2 factorial design where Facebook users were randomly assigned to see one of two ad versions (*Informational* vs. *Narrative* messages) from one of two messenger types (*Government* vs. *Academic* sources). Randomization was achieved by employing the A/B split testing functionality embedded in Facebook ads. By using A/B testing, the sample was broken down into random, non-overlapping groups (see Orazi and Johnston [40] and Kohavi et al. [12] for more details on A/B split testing). Ad campaign delivery optimization was disabled to avoid disrupting the randomization. To prevent a subject from receiving ads multiple times, the ad exposure frequency was manually capped at one over the course of the experimental period. To make sure that roughly the same number of people were in each treatment arm, we equally divided the advertising budget across treatment arms.

The target audience included Facebook adult users (age ≥ 18 yrs), living in nine wildfire smoke-prone states in the Western U.S. including Arizona, California, Colorado, Idaho, Montana, Nevada, Oregon, Utah, and Washington. The target audience excluded users who opted out of receiving Facebook ads.

We measured the CTR for each treatment arm, which quantifies the number of clicks relative to the number of times the ad was shown. CTRs (i.e., response rates) are a commonly used digital marketing metric that represents users’ engagement and motivation [12, 40, 41]. To conduct a power analysis, we ran simulations based on realistic CTRs for Facebook newsfeed ads in the North American region. The median CTR across all industries in the Facebook newsfeed in 2020 was estimated to be 1.11% [42]. We simulated the data generating process such that the interaction effect of message framing and the messenger was half the size of their main effects on CTR. The power simulations imply that, with a sample size of 1.76 million users, we could detect main effects of 0.2 percentage point increase and an interaction effect of 0.1 percentage point increase in CTR with 80% power and a Type 1 error rate of 5%.

Data were collected for 14 consecutive days in September 2021 and October 2021. The advertisement budget and duration were pre-specified. The entire campaign reached over 1.8 million users.

### Intervention materials

The informational message was a nonnarrative expository text that provided facts and reasons to support a claim about health risks associated with wildfire smoke exposure. The text was 87 words in length and was adapted from widely used PSAs about the same topic to replicate the status quo. We synthesized the text from PSAs delivered by the Centers for Disease Control and Prevention (CDC), U.S. Environmental Protection Agency (EPA), U.S. Department of Health and Human Services (HHS), and local government agencies in the study region (see Additional file 1 Fig. S1 for the screenshots of informational ads).

The narrative intervention was roughly the same length as the informational message (89 words) and embedded the same message, but in narrative form. To make the two framings comparable, the narrative intervention was deliberately framed to closely match the informational detail provided in the informational message frame. The narrative story featured Alex C., a generic character who experienced wildfire smoke inhalation impacts during the previous wildfire season. Several studies report that realism is central to narrative engagement [43–45]. Realistic narratives refer to a story-focused framing whose characters’ behaviors could reasonably occur in real world, are related to everyday experiences, portray actual events, and can fulfill the targeted audiences’ expectations. To construct a realistic digital narrative aligned with real-world stories of wildfire smoke exposure, we conducted a review of published stories via LexisNexis —an online tool with an advanced search engine enabling us to review contents published as news, blog posts, press article, etc. with the possibility of narrowing down the search to stories of those who suffered or experienced wildfire smoke. The text was adapted and synthesized from multiple sources and was based on the experience of real characters. We used a generic name for the story character to maintain confidentiality. To be inclusive, the chosen name was gender-, race-, and ethnicity-neutral with no age and location specifications (see Additional file 1 Fig. S1 for the screenshots of narrative ads). The perceived messenger on the ads was manipulated by varying the page extension between .*EDU* (for academic messenger) and .*GOV* (for government messenger).

We created a Facebook page named *Wildfire Smoke* to deliver the ads and to serve as a landing page for clickers (see Additional file 1 Fig. S2 for the screenshots of the landing page). This page provided visitors with publicly available information about wildfire smoke risk and mitigation actions that were scientifically sound (e.g., resources developed by CDC and EPA).

### Statistical analysis

We contrast the CTRs across two (message frames: narrative vs informational) by two (messengers: academic vs government) treatment combinations and estimate the effects on the CTRs from the message frame, messenger, and their interactions. Additionally, we estimate conditional treatment effects moderated by gender, the predominant political party affiliation in recipients’ region, and regional records of heavy smoke exposure (all moderators were pre-registered). These subgroup analyses are motivated by persistent claims of gender differences in affective processing and orientation (e.g., see Friesdorf et al. [46]), the previous findings about political affiliation differences in narrative receptivity [21], and the geographic heterogeneity in the historic trends of smoke exposure, which may lead users to behave differently in response to public health messages and how such messages are framed. For the gender analysis, we use Facebook’s aggregate-level ad reports (i.e., CTR data) that are broken down into fixed “gender-age” bins (i.e., males/females 18–24, 25–34, 35–44, 45–54, 55–64 and 65+ yrs. old). For the two regional attributes (i.e., smoke exposure and predominant party affiliations), we use Facebook’s aggregate-level ad reports that are broken down into designated market areas (DMAs). A DMA is a region that includes multiple adjacent counties in the U.S. and is used by media (e.g., Facebook) to define television and radio markets.

To measure the dominant political party affiliation in a DMA, we first calculated the dominant political party affiliation in the counties that make up that DMA. If the number of registered voters for the Republican Party was greater than the number of registered voters for the Democratic Party, the county was considered majority-*Republican* (data was obtained for November 2020—the most recent presidential election). We acknowledge that measuring the moderating effect of individual-level party affiliation would also be interesting, but we have no way of matching our Facebook participants to public voting databases. Smoke exposure was also first calculated at the county level. We used the methods and the dataset described in Vargo's work [47]. This method utilizes data from the National Oceanic and Atmospheric Administration’s (NOAA) Office of Satellite and Product Operations Hazard Mapping System’s Smoke Product to estimate the potential daily burden of smoke related to wildland fires on communities across the United States. For each county, we estimated the daily exposure to wildfire smoke and assigned it to one of three categories: light, medium, or heavy, approximately corresponding to fine particulate matter (PM_2.5_) concentrations of 0–10, 10–21, and 22+ μg/m^3^, respectively (see Vargo for more details [47]; also see Haikerwal et al. [48]). Then, smoky days was calculated as the number of days in the past 28 months the county population experienced heavy exposure to wildfire smoke (28 months is the national average length-of-stay of a U.S. renter). Finally, we used the method described in Carney [49] and mapped counties to DMAs by determining which counties comprised each DMA and weighting the data by county population.

We specified our main and moderator analyses and preregistered our analysis plan on Open Science Foundation prior to examining the data and conducting the analysis herein (see https://osf.io/jmwuf). To reject the null hypotheses, we used two-tailed z-tests, and to control the false positive rate in multiple comparisons, we used the Bonferroni correction with family-wise error rate of (FWER ≤0.05) [50]. We conducted six different comparisons (two main effects, one interaction effect, and three moderator effects) using an alpha level of 0.05, and thus, employing Bonferroni correction would result in new alpha level to be 0.05 divided by 6. Additionally, to increase the precision of the estimation, we used logistic regression estimators that adjustsed for covariates including gender, age, and regional characteristics such as history of smoky days and predominant political party affiliation of the region. However, to protect the confidentiality of users, Facebook does not allow the generation of reports with all of these covariates combined. Instead, one could obtain ad reports including total observed clicks and non-clicks for each treatment arm broken down into subgroups, either by fixed gender-age bins or by regions (i.e., DMAs). Therefore, regression models could not include all covariates simultaneously (see Supplemental material).

## Results

The ads were shown to 1,838,100 people. Table 1 shows the descriptive statistics of the full sample and each treatment arm. Overall, 26,390 unique users clicked on ads. Even though the frequency—the number of times an experimental condition was shown to an individual user—was manually capped at one, in some instances, Facebook showed the same ad to a user more than once. This yielded an average frequency of 1.014. This algorithmic error is endogenous to the platform and needs to be accounted for. We used the correction rule suggested by Orazi and Johnston [40]. For each treatment arm, we divided the total number of clicks by the frequency, rounding down. The corrected number of clicks and CTRs for each treatment arm is shown in Table 1.

**Table 1:** Descriptive statistics of the full sample and each treatment arm, Facebook users in Western U.S

Treatment	Framing	Informational		Narrative		All
	Messenger	Government	Academic	Government	Academic	
		*n* = 463,135	*n* = 464,754	*n* = 456,529	*n* = 453,682	*n* = 1,838,100
Outcomes	#Clicks	5825	5851	7240	7474	26,390
	Frequency	1.000	1.000	1.029	1.028	1.014
	Corrected #clicks	5825	5851	7037	7267	25,980
	Click through rate	1.26%	1.26%	1.54%	1.60%	1.41%
Age	Portion 18–24	4.5%	4.2%	5.8%	5.5%	5.0%
	Portion 25–34	22.7%	22.1%	23.7%	22.8%	22.8%
	Portion 35–44	24.2%	24.5%	23.6%	25.6%	24.5%
	Portion 45–54	20.2%	20.4%	19.7%	19.1%	19.8%
	Portion 55–64	15.5%	15.0%	15.1%	15.1%	15.2%
	Portion 65+	11.0%	11.1%	10.2%	10.7%	10.7%
Gender	Portion Female	42.7%	42.5%	43.2%	42.7%	42.8%
Predominant Party affiliation in recipients’ region	Portion Republican	25.8%	25.8%	25.2%	25.4%	25.5%
Smoky days in recipients’ region	Mean (SD)	41.4 (17.3)	41.2 (17.1)	41.5 (17.4)	41.0 (17.2)	45.7 (17.3)

Frequency is the average number of times an ad was shown to individuals. Corrected clicks = Clicks/Frequency; Click through rate = Corrected clicks/total recipients of an ad. Facebook does not provide data about users’ political party affiliation or number of days they were exposed to wildfire smokes. Facebook provides aggregate level data about how many people were shown the ads by subregions (i.e., DMAs). The predominant party affiliation and the number of smoky days were determined based on secondary data for each subregion

**Fig. 1:**
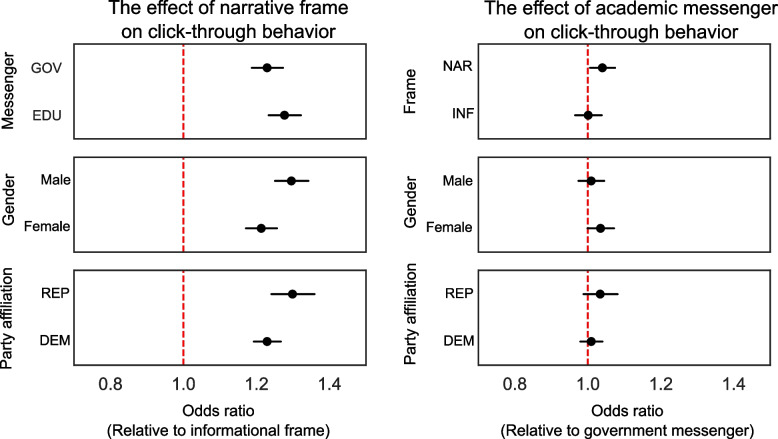
Estimated treatment effects of narrative framing, academic messenger and their interactions, conditional on gender and the predominant regional political party affiliation based upon voter registrations in the recipients’ area of residence. Error bars show 95% confidence intervals. NAR = narrative frame, INF = informational frame, EDU = academic messenger, GOV = government messenger, DEM = majority-Democrat regions, REP = majority-Republican regions

The average CTR for informational ads was 1.26%. Recipients of the narrative framing had an average CTR that was 0.3 percentage points higher than recipients of the informational framing, or a 25.3% increase (95% CI = 22.2, 28.4%, *p* < 0.001). Users who received their message from the academic messenger had an average CTR that was 0.03 percentage points higher than users who received the message from the government messenger, or a 2.2% increase (95% CI = − 0.3, 4.7%, *p* = 0.076). The average effect of the narrative framing on CTRs was 3.9% higher when the ad came from academic messenger (95% CI = − 1.1, 9.1%, *p* = 0.13) (Fig. 1). This estimated interaction effect of academic messenger and narrative framing on CTRs was small and statistically nonsignificant. Our estimated treatment effects are robust to covariate adjustment using logistic regression (Table 2).

**Table 2:** Estimated treatment effect of framing on click-through behavior

	Treatment Effects				Moderated Treatment Effects		
	No Interaction No cov. Adjustment	Interaction No cov. Adjustment	Interaction cov. Adjustment	Interaction cov. Adjustment	Conditional on Gender	Conditional on Regional Party Affiliation	Conditional on Regional Smoky Days
	(1)	(2)	(3)	(4)	(5)	(6)	(7)
Narrative	0.225***	0.206***	0.215***	0.214***	0.249***	0.198***	0.329***
	[0.2, 0.25]	[0.171, 0.241]	[0.18, 0.251]	[0.178, 0.25]	[0.205, 0.292]	[0.159, 0.237]	[0.258, 0.4]
EDU Messenger	0.022	0.001	0.003	0.006	0.003	− 0.001	0.006
	[−0.003, 0.046]	[− 0.036, 0.038]	[− 0.034, 0.04]	[− 0.031, 0.043]	[− 0.035, 0.039]	[− 0.041, 0.04]	[− 0.031, 0.044]
Narrative*EDU	–	0.038	0.032	0.022	0.032	0.022	0.021
		[−0.011, 0.087]	[−0.018, 0.082]	[− 0.028, 0.073]	[− 0.018, 0.082]	[−0.028, 0.072]	[− 0.029, 0.072]
Narrative*Female	–	–	–	–	−0.067**	–	–
					[−0.116, − 0.017]		
Narrative*Republican	–	–	–	–	–	0.053	–
						[−0.001, 0.108]	
EDU*Republican	–	–	–	–	–	0.022	–
						[−0.032, 0.077]	
Narrative*Smoky Days	–	–	–	–	–	–	−0.003***
							[−0.004, − 0.001]
Intercept	−4.374	−4.363	−4.495	−5.113	−4.514	−5.1	−5.176
Covariate: Age	No	No	Yes	No	Yes	No	No
Covariate: Gender	No	No	Yes	No	Yes	No	No
Covariate: Party Affiliation	No	No	No	Yes	No	Yes	Yes
Covariate: Reginal Smoky Days	No	No	No	Yes	No	Yes	Yes
Observations	1,838,100	1,838,100	1,801,959	1,789,380	1,801,948	1,789,380	1,789,380

Estimated treatment effect of framing on click-through behavior, and estimated treatment effects conditional on gender, regional party affiliation, and regional smoky days. The estimated effect is the change in the log odds of click-through behavior (CTB). Logistic regression estimation. Dependent variable is the CTB. The odds of CTB can be measured as CTR/(1-CTR), where CTR is the click-through rate. 95% confidence intervals in brackets. * *p* < 0.05, ** *p* < 0.01, *** *p* < 0.001

In our moderator analyses, we estimated that the narrative framing, as compared to the informational framing, increased response rates among females by 20% (95% CI = 14.1, 26.1%), while this increase was 27.7% (95% CI = 21.5, 34.3%) among males (Fig. 1). The difference in subgroup treatment effects is − 6.4% (95% CI = − 11, − 1.7%, *p* < 0.01). This difference is statistically significant using the Bonferroni correction to control for multiple comparisons in our moderator analysis.

Furthermore, we estimated that the narrative framing increased CTRs among individuals living in majority-Republican areas by 28.9% (95% CI = 23.9, 34%), while this effect was smaller in majority-Democrat areas: 21.9% (95% CI = 15.7, 28.4%) (Fig. 1). The difference in the subgroup treatment effects is 5.4% (95% CI = − 0.1, 11.4%, *p* = 0.056), but it is not statistically significant using the Bonferroni correction to control for multiple comparisons in our moderator analysis. In addition, for ads from the academic messenger, as compared to the government messenger, we estimated a small, statistically nonsignificant difference of 2.2% (95% CI = − 3.2, 8.0%, *p* = 0.42) in CTRs among majority-Republican areas relative to majority-Democrat areas.

**Fig. 2:**
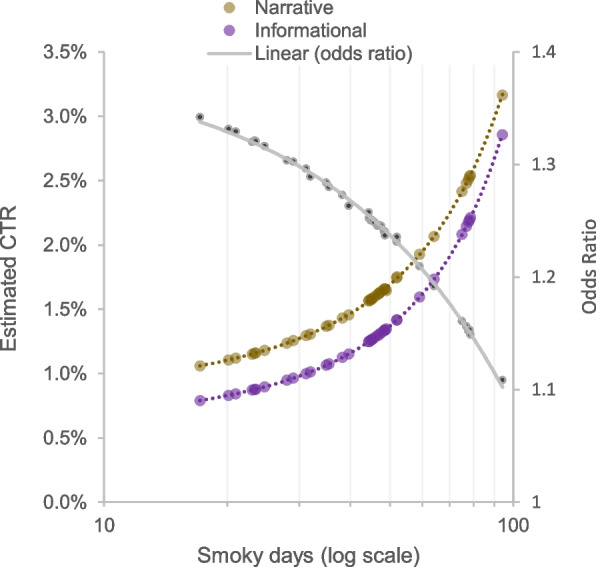
The estimated click through rate (CTR) for informational and narrative framing given the number of smoky days in the targeted areas. A logistic regression estimator is used. The second vertical axis on the right shows the odds ratio of click through behavior for narrative ads relative to informational ads. Smoky days = number of days in the past 28 months a region was exposed to heavy wildfire smoke

Finally, the effect of narrative framing on CTRs was moderated by the number of days in the past 28 months that an individual’s region was exposed to heavy wildfire smoke. We estimate that every one day increase in the number of smoky days leads to a decrease in the effect of the narrative framing of 0.3% (95% CI = − 0.4, − 0.1%, *p* < 0.001). This estimated effect implies that the effect of the narrative framing is larger among users who live in regions with less frequent exposure to heavy wildfire smoke (see Fig. 2).

## Discussion

Effective communication of health information is critical to mitigate the adverse effects of environmental health hazards, such as wildfire smoke exposure. Yet, the informational messages about environmental health hazards that are typically used in PSAs conveyed by government messengers may not be the most effective way to communicate risk. These messages often adopt a fact-based, scientific language that is not easy to comprehend and is not relatable, particularly by the most vulnerable populations including people of low socio-economic status, outdoor workers, and older adults [37, 51, 52].

Our study provides causal evidence that shifting from a fact-focused, informational framing to a story-focused, narrative framing can affect peoples’ willingness to seek out information about the hazards of wildfire smoke and the ways they can mitigate the health effects from exposure to this smoke. In our experiment, the narrative frame, in comparison to the informational frame, increased the odds of information-seeking behavior as measured by the CTR, a commonly used digital marketing metric. Our study adds to the growing literature on the effectiveness of using narratives in public health interventions. However, we did not detect a large effect from changing the messenger type from a government messenger to an academic messenger: the estimated effect was positive, but small and statistically nonsignificant.

In our study, the CTRs were higher among all subgroups of recipients of narrative ads compared to recipients of informational ads, although the estimated treatment effect was moderated by recipient and regional attributes. Narrative ads were more effective in increasing CTRs among male users compared to female users. Like the results in Byerly et al. [21], our results thus contradict prior claims that narratives are more persuasive in female subjects and that women in general may report greater narrative transportation than men [17, 53] (also see references in Byerly et al. [21]). Moreover, we find evidence that narrative framing had larger, though statistically nonsignificant, effects on CTRs among Facebook recipients living in areas where the number of registered Republicans outweighed registered Democrats. These results about the moderating effect of regional party affiliations on message framing complement prior research that looks at such moderating effect from the political affiliation of the individuals themselves [21, 34]. The results, however, are only suggestive because the estimated effect is not statistically significant after employing the Bonferroni method to control for 5% FWER in our subgroup analyses. We conclude that further research is needed before making any claims about how the effect of narrative framing varies by regional party affiliations.

Importantly, our analysis implies that narrative framing was more effective in increasing CTRs among users who live in regions where heavy smoke events are less frequent. Climate change has made human communities more prone to wildfires, especially in places with no long history of exposure to flames and smoke, where the wildfires and smoke are new-fashioned phenomena. Our results, therefore, have important implications for effective communication of wildfire smoke heath impacts and mitigation behavior, especially in such regions where wildfire smoke is mostly unheard of, and the residents are less likely to be prepared for such events.

Even though click rates on Facebook ads are often low [42], these ads can reach very large populations at low cost, thereby having the potential to induce meaningful behavior change in a population. In our experiment, the narrative framing generated 3038 additional “engaged people” (people who click on ads) over the informational framing. If the narrative framing were scaled up to the entire Facebook population in these nine states of 38 million users (an estimate from the Facebook Ads Manager platform), one could generate about 114,000 more engaged users (people who click on ads to seek more information) than one could generate if the informational framing were used instead.

Our study is an important step towards a better understanding of how best to use digital media to communicate risks and mitigation actions, an understanding that could be applied across a wide array of domains. Digital media allows for low-cost and rapid dissemination of information. Social digital media, like Facebook, have become ubiquitous in American life. According to the Pew Research Center, as of 2021, Facebook is the largest social media platform used by Americans, with more than 200 million active users across demographic groups. Facebook and other online platforms could be used to deliver, for example, personalized information on air quality conditions and health risks. Using these online platforms to conduct online randomized controlled experiments to test the behavioral effects of health communication interventions and in a wide range of contexts, like wildfire smoke safety, can potentially enable accelerated public health improvements [12]. With a better understanding of how to harness these online platforms to enhance the communication of risks and to inform the adoption of individual or community level health-protective actions, practitioners could nudge people towards behaviors that improve public health across a broad range of communities.

### Limitations

Our study has three main limitations. First, although we measured actual behavior in our randomized natural field experiment, the behavioral outcome was limited to information-seeking behavior measured by CTRs, as opposed to mitigation actions. Given the anonymized data on the Facebook platform, it was not possible to identify the subjects who observed the ads or follow-up with them about their attitudes toward, or engagement with, mitigation actions that may occur over long time periods. Yet, inducing information-seeking behavior is considered an important first step for individuals to pursue risk mitigation actions [9, 10].

Second, our design intentionally did not employ the use of named governmental or academic messengers. We avoided using specific institutions on the messages because we worried that recipient perceptions of particular institutions could confound our attempt to identify a generalizable treatment effect from changing the messenger from a government organization to an academic organization (e.g., an unknown federal agency versus an internationally known academic institution). Instead, we manipulated the landing page’s address presented on the ads between .*GOV* and .*EDU* extensions, which represented a government and an academic organization, respectively. However, these differences in the perceived messenger were potentially not salient to all ad recipients, particularly those who are not aware of domain extensions and their association with organization types. Future research can consider ways to signal differences in messenger types in a more salient way, as well as explore a wider range of institution categories, such as non-governmental organizations or health care providers.

Third, given the restrictions imposed by using Facebook ads manager, no data were made available with which we could run analyses of subgroup effects by party affiliation and smoke exposure at the county level. Our pre-analysis plan had specified doing the analysis at this level, but after learning more about the Facebook system, we determined that we could only do the analysis at a higher level of aggregation (i.e., DMAs.). This is the only deviation from our registered pre-analysis plan.

## Conclusion

We conducted a natural field experiment on a social media platform to study how changes in the framing and messenger of public health risk messages affect behavior. Specifically, we contrasted the use of narrative vs information framing and the use of academic vs government messengers, and their interactions, on information-seeking behavior to avert the public health impacts of increased wildfire smoke exposure. Our study fills important gaps in the empirical literature that reports on the use of online randomized controlled experiments to assess the behavioral effects of digital message framing to communicate about public health risks.

## Supplementary Information

**Additional file 1.**

## Data Availability

All data and code supporting the findings of this study are available and can be downloaded from OSF at https://osf.io/9ax6n/?view_only=55488a95825444aa830eadbd38fe3565.
