# Supplementary material for: The choice of message and messenger to drive behavior change that averts the health impacts of wildfires: an online randomized controlled experiment
Source: BMC Public Health. 2022 Dec 16;22:2359. doi: 10.1186/s12889-022-14801-6 (PMC9756613; doi:10.1186/s12889-022-14801-6)
Supplement: Supplementary file 1 — Additional file 1. [file 12889_2022_14801_MOESM1_ESM.docx]

Supplementary Materials for

**The choice of message and messenger to drive behavior change that averts the health impacts of wildfires: an online randomized controlled experiment**

**1- Supplementary Methods**

**Treatments:**

We implemented a pre-registered, online, randomized controlled, message-framing experiment using a 2x2 factorial design. We used a 2x2 design which resulted in four different treatments, in which Facebook users from a set of nine states in the U.S. were randomly assigned to see ads with one of two message frames (Informational vs. Narrative messages) from one of two messengers (Government vs. Academic messengers):

1. Informational message from Government (INF/GOV) (*the status quo*)
2. Informational message from Academia (INF/EDU)
3. Narrative message from Government (NAR/GOV)
4. Narrative message from Academia (NAR/EDU)

We quantify the differences in “Click Through Rates” (CTR: the rate at which users click on the ad after being exposed to it) for each of the four treatment arms, so that we can estimate the message frame effect (INF vs. NAR), the messenger effect (GOV vs. EDU), and the interaction effect of message frame and messenger. See below for the text used in different message frames:

- ***Informational frame*** (87 words)***:*** Wildfire smoke is a major risk if you are not prepared. It can even affect healthy people. Besides affecting outdoor air quality, smoke can easily travel indoors. Wildfire Smoke is a complex mixture of gases and fine particles that can penetrate deep into your respiratory system and cause health problems ranging from burning eyes and a shortness of breath to aggravated chronic heart and lung diseases. Exposure to fine particles can be linked to premature death. There are no/low-cost ways to protect yourself from wildfire smoke hazards.
- ***Narrative frame*** (89 words)***:*** Wildfire smoke is scary, even for healthy people like Alex C. who always shelters indoors when smoke is in the distance, but smoke can also easily travel indoors. Last year Alex was home during a smoky day and started to feel dizzy. Looking in the mirror, the face looking back was purple. Alex stopped breathing and was rushed to the hospital with coughing and serious chest pain, a near death experience that ended up in taking daily medication. There are no/low-cost ways to protect yourself from wildfire smoke hazards.

Also, see Fig. S1 for the screenshots of Facebook ads in our experiment.

**Facebook reports by subgroups (Breakdowns):**

Facebook identifies the geographic location of its users and requires users to provide their age and gender. This information enables Facebook to generate “breakdown” summary reports. Breakdown reports include demographics (e.g., age, gender, region) and can be applied to a list of all ad performance parameters (e.g., ***Reach***= # people who exposed, ***Clicks*** = # people who exposed and clicked, ***Non-Clicks*** = # people who exposed and did not click). For example, we can produce a summary report of “Clicks” in each treatment arm by female subgroup or by a specific geography (e.g., Los Angeles, CA). Hence, “Breakdowns” allow us to estimate subgroup treatment effects by age, gender, and location. Except for gender and age, breakdowns cannot be combined; Facebook offers ad reports broken down into subgroups by fixed “gender-age” bins (i.e., Males/Females 18-24, 25-34, 35-44, 45-54, 55-64 and 65+ yrs. old); however, it does not allow advertisers to break the results down to combined age-gender-region bins. We used subgroup reports to create a dataset for the ***Clicks*** and ***Non-clicks*** across treatments and separately for possible subgroups. This dataset enabled running a logistic regression that was used to return a significance test for the odds ratios.

**Regression analysis:**

Our dependent variable is “click through behavior”, $CTB$. $CTB$ is a dummy variable equal to one when the Facebook user clicks on an ad and zero, otherwise. The probability that a user clicks on an ad is defined as $\pi=Pr(CTB=1)$, which can be measured by the ad’s click-through rate (CTR = Clicks/Reach) for the subgroup to which the individual belongs. We use the below logistic regression model to estimate the treatment effects:

$$ln\left( \frac{\pi}{1-\pi} \right)=\beta_{0}+\beta_{1}NAR+\beta_{2}EDU+\beta_{3}NAR\times EDU+\beta_{4}X$$

Where $ln\left( \frac{\pi}{1-\pi} \right)$ is log-odds of $CTB$, $NAR$ is a dummy variable equals to 1 if the ad uses narrative frame and 0 otherwise, $EDU$ is a dummy variable equals to 1 if the ad uses academic messenger and 0 otherwise, and $X$ is the vector of control variables. Converting the coefficients ($\beta_{1}, \beta_{2},\beta_{3})$into odds ratios yields estimated treatment effects of the main treatments (NAR, EDU) and their interaction. We could obtain ad reports for each treatment arm broken down into subgroups either by fixed gender-age bins or by sub-regions (i.e., DMAs). Thus, the control variables included in the regression model could be either “age and gender” or sub-region characteristics such as “smoke exposure and the predominant party affiliation,” but not all combined.

**Moderator Analyses:**

***Gender (Individual attribute)***

We estimated separate treatment effects for female subgroups using the following regression:

$$ln\left( \frac{\pi}{1-\pi} \right)=\beta_{0}+\beta_{1}NAR+\beta_{2}EDU+\beta_{3}NAR\times EDU+\beta_{4}Female+\beta_{5}NAR\times Female+\beta_{6}Age$$

Where $Female = 1$ for users in “female” subgroups. We estimated $\beta_{5}$ to test if gender moderates the effect of narrative frame on click-through behavior.

***Political party affiliation (Regional attribute)***

We estimated separate treatment effects for subregions where the number of registered Republicans outweighed registered Democrats using the following regression:

$$ln\left( \frac{\pi}{1-\pi} \right)=\beta_{0}+\beta_{1}NAR+\beta_{2}EDU+\beta_{3}NAR\times EDU+\beta_{4}REP+\beta_{5}NAR\times REP+\beta_{6}EDU\times REP+\beta_{6}Smoke$$

Where $REP=1$ if users are located in a region, where the number of voters registered for the Republican Party was greater than the number of voters registered for the Democratic Party, and $REP=0$ otherwise, and $Smoke$ is a variable showing the history of smoke exposure in a region. We estimated $\beta_{5}$ to test if predominant party affiliation in a region moderates the effect of narrative frame on click-through behavior. In addition, we estimated $\beta_{6}$ to test if predominant party affiliation in a region moderates the effect of academic messenger on click-through behavior.

***Smoke exposure (Regional attribute)***

We estimated the moderation effect of smoke exposure using the following regression:

$$ln\left( \frac{\pi}{1-\pi} \right)=\beta_{0}+\beta_{1}NAR+\beta_{2}EDU+\beta_{3}NAR\times EDU+\beta_{4}Smoke+\beta_{5}NAR\times Smoke+\beta_{6}REP$$

We estimated $\beta_{5}$ to test if history of smoke exposure moderates the effect of narrative frame on click-through behavior.

**2- Supplementary Figures**


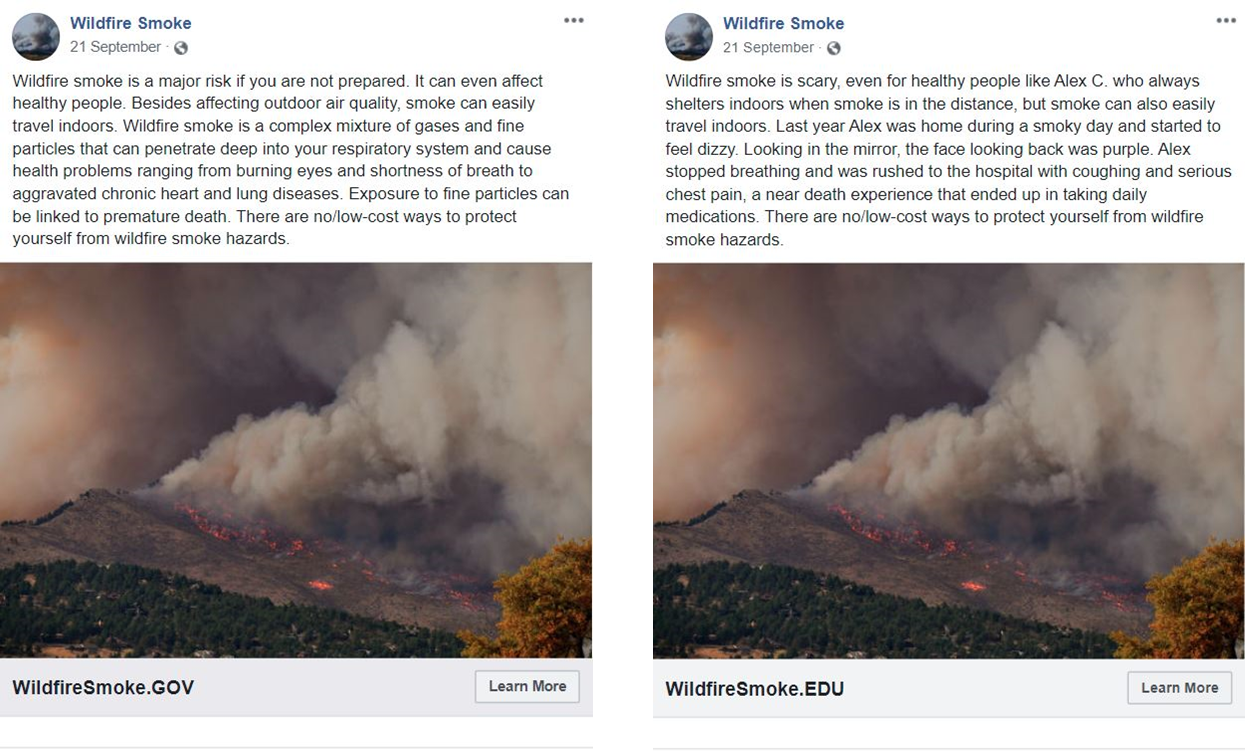


Figure S1. The screenshots of Facebook ads in our experiment. The image on the left is the screenshot of an informational ad with government messenger. The image on the right is the screenshot of a narrative ad with academic messenger.


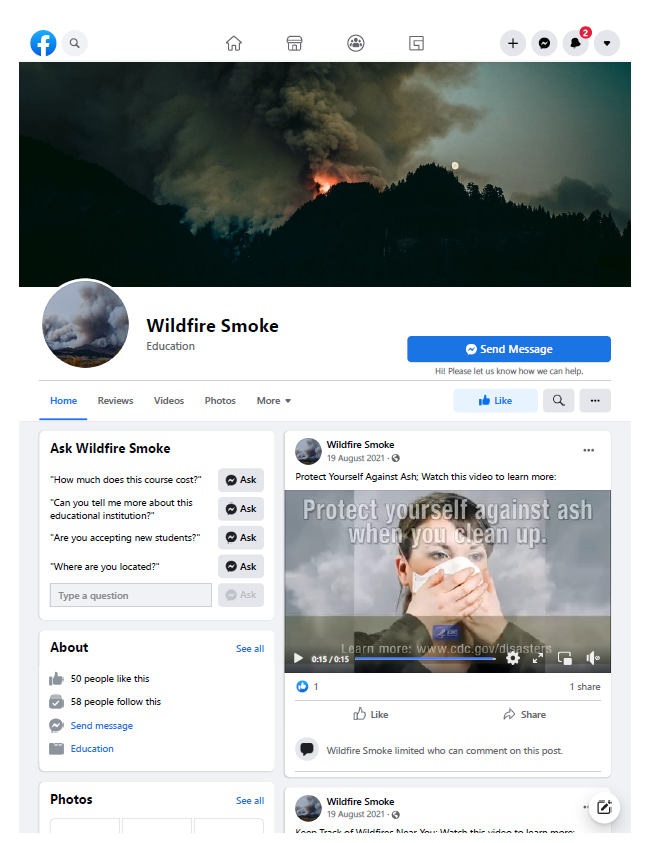


Figure S2. The screenshots of Facebook landing page. The landing page materials were developed by the U.S. Centers for Disease Control and Prevention (CDC), U.S. Environmental Protection Agency (EPA), American Lung Association (ALA), AirNow, and The United States Department of Agriculture (USDA). The information on these websites is not subject to copyright, is in the public domain, and may be freely used or reproduced without obtaining copyright permission. The use of the material, including any links to the materials on the CDC, EPA, ALA, USDA or AirNow websites, does not imply endorsement by these agencies of our work. The materials are also available on the agency websites for no charge. Original links were provided, and subjects were referred to the original websites for more information.
